# Efficacy and Resistance of Afatinib in Chinese Non-Small Cell Lung Cancer Patients With *HER2* Alterations: A Multicenter Retrospective Study

**DOI:** 10.3389/fonc.2021.657283

**Published:** 2021-05-07

**Authors:** Zhengbo Song, Dongqing Lv, Shiqing Chen, Jianhui Huang, Liping Wang, Shuguang Xu, Huafei Chen, Guoqiang Wang, Quan Lin

**Affiliations:** ^1^ Department of Clinical Trial, The Cancer Hospital of the University of Chinese Academy of Sciences (Zhejiang Cancer Hospital), Hangzhou, China; ^2^ Institute of Basic Medicine and Cancer (IBMC), Chinese Academy of Sciences, Hangzhou, China; ^3^ Department of Respiratory Disease, Taizhou Hospital, Taizhou, China; ^4^ The Medical Department, 3D Medicines Inc, Shanghai, China; ^5^ Department of Medical Oncology, Lishui Center Hospital, Lishui, China; ^6^ Department of Medical Oncology, Baotou Cancer Hospital, Baotou, China; ^7^ Department of Respiratory Disease, Ningbo Medical Center, Lihuili Eastern Hospital, Ningbo, China; ^8^ Department of Thoracic Disease Center, Rongjun Hospital, Jiaxing, China; ^9^ Department of Pulmonary Medicine, The First Affiliated Hospital of Wenzhou Medical University, Wenzhou, China

**Keywords:** non-small cell lung cancer (NSCLC), *HER2*, afatinib, efficacy, resistance

## Abstract

**Background:**

Non-small cell lung cancer (NSCLC) patients with *HER2* mutations and amplification may benefit from HER2-targeted therapy, including afatinib. However, the data regarding the clinical activity of afatinib in Chinese patients with NSCLC harboring *HER2* alterations are limited.

**Patients and methods:**

We retrospectively included metastatic NSCLC patients harboring *HER2* alterations who treated with afatinib. The clinical outcomes included overall response rate (ORR), progression-free survival (PFS) and overall survival (OS). The genomic profiling data after progression on afatinib were analyzed.

**Results:**

We included 54 patients harboring *HER2* mutations and 12 patients harboring *HER2* amplification. The ORR was 24% (95% CI, 16–36%), the median PFS was 3.3 months (95% CI, 2.2–4.4), and the median OS was 13.9 months (95% CI, 11.4–16.5). Patients with *HER2* exon 20 mutations had numerically worse ORR (17% *vs* 42%), shorter PFS (2.6 *vs* 5.8 months, HR, 2.5; 95% CI, 1.2–5.5; *P* = 0.015) and OS (12.9 *vs* 33.3 months, HR, 4.4; 95% CI, 1.3–14.8; *P* = 0.009) than patients with other mutations. For HER2-amplified patients, the ORR was 33% (95% CI, 14–61%), the median PFS was 3.3 months (95% CI, 2.6–4.0), and the median OS was 13.4 months (95% CI, 0–27.6). The most frequently mutated genes in afatinib-resistant patients were *TP53* (44%) and *EGFR* (33%). Three afatinib-resistant patients harbored secondary *HER2* alterations.

**Conclusions:**

Our results suggest that afatinib has a promising anti-tumor activity in patients with NSCLC harboring *HER2* alterations. To our knowledge, this is the largest retrospective study about the clinical activity of afatinib in NSCLC patients with *HER2* alterations.

## Introduction

Lung cancer is one of the most common malignant tumors, causing approximate 25% of the total cancer-related deaths (1). About 85% of patients with lung cancer are histologically diagnosed as non-small cell lung cancer (NSCLC) (2). Several driver genes alterations, including *EGFR* (epidermal growth factor receptor) activating mutations, *ALK* (anaplastic lymphoma kinase) rearrangement, *ROS1* (repressor of silencing 1) fusions, *BRAF* (B-Raf proto-oncogene, serine/threonine kinase) mutations, *MET* (MET proto-oncogene, receptor tyrosine kinase) alterations, and *RET* (ret proto-oncogene) fusions, are frequently detected in the patients with NSCLC (3). Targeted therapies based on these genes have been approved by the Food and Drug Administration (FDA), changing the treatment of NSCLC (4).

Human epidermal growth factor receptor 2 (*HER2*, also known as *ERBB2*) is a cancer driver gene, and 1.7–3% of NSCLC patients harbor *HER2* mutations (5–7). Most *HER2* mutations in NSCLC are present in exon 20, such as Y772_A775dup and G778_P780dup. In addition, *HER2* gene amplification occurs in 3 to 14.3% of lung adenocarcinomas (7–9). *HER2* activating mutations and amplification may activate tyrosine kinase and downstream signaling pathways, therefore conferring sensitivity to HER2-targeted therapy, such as trastuzumab, ado-trastuzumab (T-DM1) and tyrosine kinase inhibitors (TKIs). At present, T-DM1 is the only recommended HER2-targeted inhibitor for *HER2*-mutated NSCLC patients by National Comprehensive Cancer Network (NCCN) Guidelines, with an overall response rate (ORR) of 44% (10). However, no *HER2*-targeted therapy has been approved for patients with NSCLC harboring *HER2* mutations or amplification.

Afatinib is an irreversible ERBB family inhibitor, which has been approved for *EGFR*-mutated lung cancer and become one of the most common therapy in NSCLC patients. In a phase II trial with 13 advanced NSCLC with *HER2* exon 20 mutations, the overall response rate (ORR) of afatinib as second-line treatment was 7.7% and the median progression-free survival (PFS) was 15.9 weeks (11). Several retrospective trials revealed better activity of afatinib in patients with *HER2* exon 20 mutations, with an ORR from 13 to 33% (5, 12–15). However, the interpretation of the results from all these studies were limited by the small sample sizes. In addition, the efficacy of HER2-TKI in patients with *HER* mutations besides *HER2* exon 20 mutations and *HER2* amplification has been rarely studied. Seven patients with other *HER2* mutations except exon 20 mutations were enrolled into the phase II trial of T-DM1, and two of these patients had a partial response, with a S310F (exon 8) mutation and a V659E (exon 17) mutation, respectively (10). Another research showed that three of four NSCLC patients with V659E or G660R (exon 17, located in transmembrane domain) achieved responses from afatinib treatment (16).

Herein, we conducted a multicenter, retrospective study to analyze the anti-tumor activity of afatinib in patients with NSCLC harboring *HER2* alterations including mutations and amplification. Furthermore, we tried to explore the potential secondary resistant mechanisms of afatinib by next generation sequencing (NGS). We present the following article/case in accordance with the STROBE reporting checklist.

## Methods

### Patients and Study Design

This multicenter, retrospective study included patients with non-small cell lung cancer (NSCLC) harboring *HER2* alteration treated with afatinib between May 2015 and July 2019, from Zhejiang Cancer Hospital, Taizhou Hospital, Baotou Cancer Hospital, Lihuili Eastern Hospital and Rongjun Hospital. Eligible patients were 18 years or older, and had a diagnosis of stage IV NSCLC, a *HER2* alteration, measurable disease as per investigator-assessed Response Evaluation Criteria in Solid Tumors (RECIST), v1.1. Patients received afatinib at a dose of 40 mg daily until disease progression or intolerable adverse events. This retrospective study was approved by the Institutional Review Board Committee of Zhejiang Cancer Hospital. Research was conducted in accordance with the 1964 Declaration of Helsinki and its later amendments. The informed consent was waived because of the retrospective nature of this study.

### Data Collection and Response Assessment

Baseline clinical information were collected from electronic medical records, including age, sex, Eastern Cooperative Oncology Group (ECOG) performance status, tumor histology, smoking status, *HER2* alteration subtype, and afatinib treatment line. These clinical data were verified independently by two oncologist physicians. Tumor size measurement according to radiologic imaging was conducted by radiologists. Best response was determined according to Response Evaluation Criteria in Solid Tumors (RECIST, v1.1). The outcomes were ORR, PFS, and overall survival (OS). ORR was defined as the proportion of patients who have a partial response (PR) or complete response (CR). PFS was defined as the time interval from initial afatinib treatment to progression or death from any cause. OS was defined as the duration from the beginning of afatinib treatment to death from any cause.

### Molecular Testing

The baseline *HER2* gene alterations were tested by NGS in an accredited local laboratory (for example as shown in **Figure S1**). Genomic profiling when progression on afatinib treatment was tested in a CLIA-accredited/CAP-certified laboratory (3D Medicines Inc., Shanghai, China). The NGS panel targeted cancer-related genes was performed on the NextSeq500 platform (Illumina, CA, USA) (17). DNA extracts (30–200 ng) were sheared to 250 bp fragments using an S220 focused-ultrasonicator (Covaris). Libraries were prepared using the KAPA Hyper Prep Kit (KAPA Biosystems) following the manufacturer’s protocol. The captured libraries were loaded onto a NextSeq500 platform for 100 bp paired-end sequencing with a mean sequencing depth of 500×.

Raw data of paired samples (an FFPE sample and its normal tissue control) were mapped to the reference human genome hg19 using the Burrows–Wheeler Aligner (v0.7.12). PCR duplicate reads were removed and sequence metrics were collected using Picard (v1.130) and SAMtools (v1.1.19), respectively. Variant calling was performed only in the targeted regions. Somatic single nucleotide variants (SNVs) were detected using an in-house developed R package to execute a variant detection model based on binomial test. Local realignment was performed to detect indels. Variants were then filtered by their unique supporting read depth, strand bias, base quality as previously described. All variants were then filtered using an automated false positive filtering pipeline to ensure sensitivity and specificity at an allele frequency (AF) of ≥1%. Single-nucleotide polymorphism (SNPs) and indels were annotated by ANNOVAR against the following databases: dbSNP (v138), 1000Genome and ESP6500 (population frequency >0.015). Only missense, stopgain, frameshift and non-frameshift indel mutations were kept. Copy number variations (CNVs) and gene rearrangements were detected. The interpretation of variants were based on American College of Medical Genetics and Genomics (ACMG) standards and guidelines.

### Statistical Analyses

All statistical analyses were conducted using the SPSS statistical package, version 20.0 (SPSS Inc®, Chicago, Illinois, USA) and GraphPad prism v6 (GraphPad, La Jolla, CA, USA). The PFS and OS were estimated by Kaplan–Meier curves, with *P* value determined by a log-rank test. And we calculated hazard ratio (HR) and its 95% confidence intervals (CIs) by Cox regression. Univariate and multivariate analyses were performed by Cox proportional hazard model. A two-sided *P* <.05 was considered statistically significant.

## Results

### Baseline Characteristics

A total of 66 patients with lung cancer were included in this retrospective study. The baseline characteristics are shown in **Table 1**. The median age was 59 years (range, 30–81), and 65% (43/66) were female. Eight patients (12%) had an Eastern Cooperative Oncology Group (ECOG) performance status of 2 and the rest were ECOG 0–1. Most patients were adenocarcinoma (92%, 61/66) and non-smokers (67%, 44/66). Ten (15%) patients had brain metastases. All the patients received afatinib as a single agent. The median line of afatinib treatment was 2 (range, 1–7). Twenty-four patients (36%, 24/66) received afatinib as first-line therapy, and 42 patients (64%, 42/66) as second-line or beyond therapy (**Table 1**). The median follow-up period was 13.9 months (range: 2.1–39.5).

**Table 1:** Baseline characteristics.

Sex, n (%)	
Male	23 (35%)
Female	43 (65%)
Age, years	
Median (range)	59 (30–81)
ECOG performance status, n (%)	
0	12 (18%)
1	46 (70%)
2	8 (12%)
Histology, n (%)	
Adenocarcinoma	61 (92%)
Squamous carcinoma	4 (6%)
Adenosquamous carcinoma	1 (2%)
Smoking status, n (%)	
Yes	21 (32%)
No	44 (67%)
Unknown	1 (2%)
Number of metastases	
Median (range)	2 (1–7)
Previous treatments	
Chemotherapy	39 (59%)
Bevacizumab	4 (6%)
TKI	3 (5%)
Anti-PD-1/L1 inhibitors	2 (3%)
Afatinib treatment line, n (%)	
First	24 (36%)
Second or beyond	42 (64%)
*HER2* alterations, n (%)	
* HER2* mutations	54 (82%)
exon 20 mutations	42
other mutations	12
* HER2* amplification	12 (18%)

ECOG, Eastern Cooperative Oncology Group; HER2, Human epidermal growth factor receptor 2; TKI, tyrosine kinase inhibitor.

**Figure 1:**
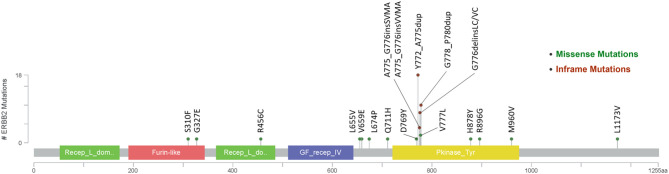
*HER2* mutational map.

Fifty-four patients (82%) harbored mutations in *HER2* gene (**Figure 1**), most of which were identified in exon 20 (78%, 42/54). In addition, twelve patients carried *HER2* amplification. Among the patients with *HER2*-mutated lung cancer, the most common mutation was Y772_A775dup (33%, 18/54), followed by G778_P780dup (19%, 10/54) and G776delinsVC/LC (15%, 8/54).

### Clinical Activity of Afatinib in NSCLC Patients With HER2 Alterations

**Figure 2:**
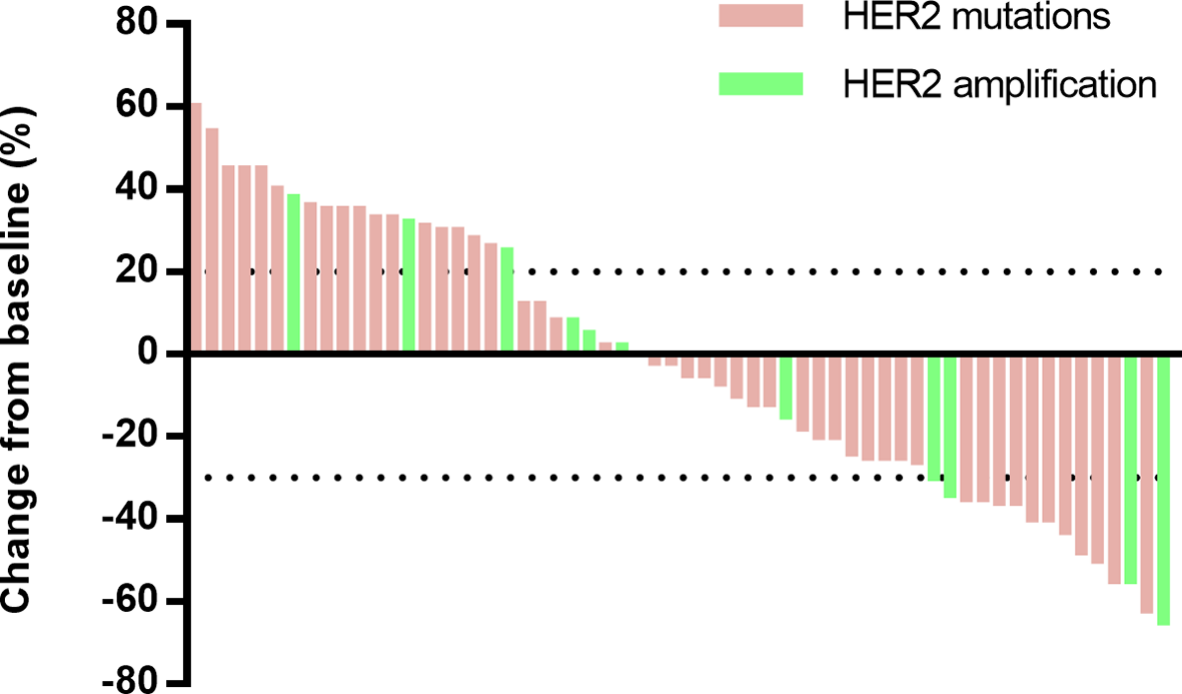
Maximum percentage change from baseline in target lesions.

The responses to afatinib were evaluated according to RECIST 1.1 (**Figure 2**), and the best response to afatinib was partial response (PR) in 16 patients (24%), stable disease (SD) in 24 patients (36%), and progressive disease (PD) in 26 patients (39%, **Table 2**). The ORR was 24% (95% CI, 16–36%), and disease control rate (DCR) was 61% (95% CI, 49–72%).

**Table 2:** Clinical activity of afatinib in NSCLC patients with *HER2* alterations.

Variable
Best response, n (%)
Partial response	16 (24%)
Stable disease	24 (36%)
Progressive disease	26 (39%)
Overall response rate, %	24%
Disease control rate, %	61%
Progression-free survival	
Events, n (%)	62 (94%)
Median, months (95% CI)	3.3 (2.2–4.4)
Overall survival	
Events, n (%)	40 (61%)
Median, months (95% CI)	13.9 (11.4–16.5)

CI, confidence interval.

**Figure 3:**
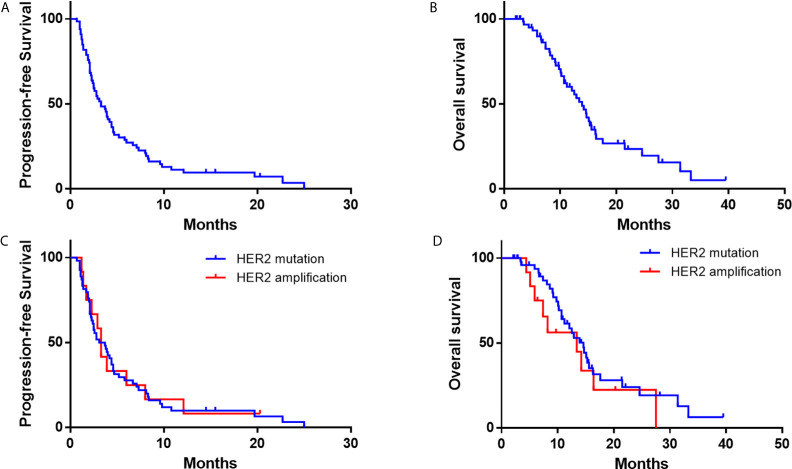
Kaplan–Meier estimates of progression-free survival and overall survival of NSCLC Patients with *HER2* alterations. **(A, B)** progression-free survival and overall survival of the whole cohort; **(C, D)** progression-free survival and overall survival according to *HER2* mutations or amplification.

As data cutoff, 62 (94%) out of 66 patients had died or had disease progression and 40 (61%) patients had died. The median PFS was 3.3 months (95% CI, 2.2–4.4), and the median OS was 13.9 months (95% CI, 11.4–16.5) (**Table 2** and **Figure 3**). The median duration of response was 7.1 months (95%CI, 6.4–7.7 months). Furthermore, we deeply analyzed the efficacy of afatinib in patients with different *HER2* alterations. Among the patients with a *HER2* mutation, the ORR, mPFS and mOS were 22%, 3.4 months (95% CI, 1.4–4.7) and 14.6 months (95% CI, 11.6–17.6), similar with the whole cohort (**Table 3**). In addition, four of the patients with *HER2* amplification (33%, 4/12) achieved a PR (**Figure 2**). The mPFS and mOS of these patients were respectively 3.3 months (95% CI, 2.6–4.0) and 13.4 months (95% CI, 0–27.6), comparable to those of the patients with a *HER2* mutation (**Figures 3C, D**).

**Table 3:** Clinical Activity of Afatinib according to *HER2* alterations.

*HER2* alterations	ORR, %	mPFS, month (95%CI)	mOS, month (95%CI)
Mutations	22%	3.4 (1.4–4.7)	14.6 (11.6–17.6)
exon 20 mutations	17%	2.6 (0.9–4.1)	12.9 (8.8–17.0)
Y772_A775dup	33%		
G778_P780dup	10%		
G776delinsVC/LC	0		
775_G776insSVMA	0		
A775_G776insVVMA	0		
V777L	0		
other mutations	42%	5.8 (0–10.1)	33.3 (1.2–65.5)
Amplification	33%	3.3 (2.6–4.0)	13.4 (0–27.6)

CI, confidence interval; HER2, Human epidermal growth factor receptor 2; mOS, median overall survival; mPFS, median progression-free survival; ORR, overall response rate.

Since *HER2* exon 20 mutation is the most common mutation for *HER2* in patients with NSCLC, we further compared the outcomes of patients with exon 20 mutation and other mutations. As for *HER2* exon 20 mutations, the total ORR was 17%, and the ORRs of the patients with Y772_A775dup mutation and G778_P780dup were 33 and 10%, respectively, while the ORR was 0% in patients with other exon 20 mutations including G776delinsVC/LC, A775_G776insSVMA, A775_G776insVVMA, and V777L (**Table 3**). In patients with other *HER2* mutations, five (42%) out of 12 patients achieved a PR (L655V [exon 17], H878Y [exon 21], R896G [exon 22], M960V [exon 24] and L1173V [exon 27]). Patients with *HER2* exon 20 mutations had worse PFS (2.6 *vs* 5.8 months, HR, 2.5; 95% CI, 1.2–5.5; *P* = 0.015) and OS (12.9 *vs* 33.3 months, HR, 4.4; 95% CI, 1.3–14.8; *P* = 0.009) than patients with other *HER2* mutations (**Figure S2**).

We also performed subgroup analysis according afatinib treatment lines. The ORR was 42% in patients who received afatinib as first-line treatment compared with 14% in those who received afatinib as secondary-line or beyond treatment. Patients who received afatinib as secondary-line or beyond treatment had shorter PFS and OS compared with patients who received afatinib as first-line treatment (mPFS = 2.7 *vs* 4.7 months; OS = 11.2 *vs* 15.6 months; **Figure S3**). Multivariate analysis showed that afatinib treatment line and brain metastasis were associated with PFS (*P* = 0.026 and 0.017, respectively), and ECOG performance status was associated with OS (*P* = 0.046) (**Tables S1** and **S2**).

### Potential Biomarkers for Resistance to Afatinib

To reveal potential biomarkers of resistance to afatinib, NGS was performed from blood or tissue samples of nine patients after progression on afatinib treatment. Pathogenic and likely pathogenic mutations were analyzed. We observed most patients (78%, 7/9) still harbored *HER2* alterations after afatinib treatment (**Table S3**). Among these patients, three patients harbored secondary *HER2* alterations (p.G776delinsLC [patient 3], Y772_A775dup [patient 4], and amplification [patient 6], **Table S3**). The most frequently mutated genes in afatinib-resistant patients were *TP53* (44%) and *EGFR* (33%). Besides, one patient carried a *NRAS* mutation and another patient had no *HER2* alteration nor other pathogenic mutation when progression on afatinib (**Table S3**).

## Discussion

In the present study, afatinib showed promising anti-tumor activity in patients with NSCLC harboring *HER2* alterations including *HER2* exon 20 mutations, other mutations and *HER2* amplification. To our knowledge, this is the largest retrospective study on clinical activity of afatinib in NSCLC patients with *HER2* alterations.

Most previous studies focused on *HER2* exon 20 insertions. Recent studies reported that NSCLC patients with *HER2* exon 20 insertions had an ORR of 13–19% from afatinib treatment (14, 15, 18). The sole to date prospective study (11) on afatinib in NSCLC patients with *HER2* exon 20 insertions only enrolled 13 patients, with a modest clinical outcomes (ORR = 7.7%). In the largest cohort of NSCLC patients with *HER2* exon 20 insertions, Mazières et al. (12) reported clinical activity of chemotherapy and HER2-targeted drugs. The ORR of the patients (*N* = 29) treated with TKIs (neratinib, afatinib, and lapatinib) was 7.4%. Among the patients (*N* = 11) who were treated with afatinib, the ORR was 18.2%. In the present study, most *HER2* mutations were exon 20 insertions (61%), which was similar with previous studies. We observed an ORR of 17% in these patients, which was comparable with previous retrospective studies. Of the patents with most common Y772_A775dup mutation, ORR was 33%, which suggests that these patients might have better clinical outcome from afatinib.

Moreover, we found *HER2* other mutations except exon 20 mutations were also sensitive to afatinib. Five (42%) of these patients achieved response from afatinib treatment. Among these patients who were response to afatinib, one patient with a L655V (exon 17) mutation had a PFS of 8.1 months. L655V (exon 17) is located in transmembrane domain (TMD) that is important to stabilize the active *HER2* homodimer (19). And L655V is close to V659/G660, which were demonstrated to be sensitive to afatinib (16). One lung squamous cell carcinoma patient with a *HER2* R896G (exon 22) mutation had a long PFS of 14.5 months, which was recently reported as a case report (20). Another patient with a M960V (exon 24) mutation received afatinib as third-line therapy, and achieved a PR and a PFS of 7.1 months. The other two patients respectively harbored H878Y (exon 21) and L1173V (exon 27), and the PFS were 22.7 and 25.0 months, respectively. These results suggest that the patients with *HER2* other mutations except exon 20 mutations could also benefit from HER2-targeted inhibitors.

So far, the standard care for NSCLC patients with *HER2* amplification is chemotherapy. Although T-DM1 is recommended by NCCN Guidelines for *HER2*-mutated NSCLC patients, no *HER2*-targerd inhibitors are approved for NSCLC patients with *HER2* mutations or amplification. In a phase II trial of dacomitinib in lung cancer patients with *HER2* alterations, none of four patients with *HER2*-amplified tumors responded (21). Recently, two studies on *HER*-mutated NSCLC patients treated with pyrotinib, a pan-HER inhibitor, showed that ORRs were 53.3 and 30%, and mPFSs were 6.4 months and 6.9 months, respectively (22, 23). An *in vitro* study and phase II trial demonstrated another pan-HER inhibitor poziotinib had potent clinical activity against *HER2* mutations (24, 25). In breast cancer, gastric Cancer, and colorectal cancer, *HER2* amplification was demonstrated to be associated with the clinical outcomes of HER2-targeted treatment (26, 27). In this study, we presented an ORR of 33% in the NSCLC patients with *HER2* amplification, and this is the first time that clinical activity of afatinib in *HER2*-amplified NSCLC patients has been reported. These results indicate HER2-targeted treatment might be one of the choices for these patients.

Primary and acquired resistance is the main reason for progression disease when patients received TKIs treatment. Currently, we know much about the mechanisms for resistance of EGFR-targeted treatment, but researches about resistance to HER2-targerted inhibitors in NSCLC patients are lacking. Chuang et al. (13) suggested PIK3CA mutation and *HER2* gene amplification may be the potential mechanisms for resistance during HER2-targeted treatment. However, the results were analyzed from four cases, which is hard to reach statistical significance. Herein, we performed NGS for nine patients when progression on afatinib treatment. Of three patients harbored secondary *HER2* alterations, two carried a *HER2* exon 20 insertion and another carried *HER2* amplification as secondary alteration. Previous studies demonstrated that secondary ALK mutations could induce resistance of ALK inhibitors (28, 29). Whether *HER2* secondary alterations resistance mechanism to afatinib need to be determined in further studies. In addition, we found *TP53* was recurrently mutated (44%) in afatinib-resistant patients. Several studies reported that *TP53* mutations were associated with inferior clinical effect of EGFR-targeted inhibitors (30–32). One patient harbored *TP53* and *RB1* co-mutations, which were associated with an increasing risk for small cell transformation and resistance to TKIs treatment (33–36).

This study still has several limitations. Firstly, we cannot completely avoid the reporting bias because of this work’s retrospective nature. Secondly, due to a lack of control arm, comparison with other therapies was not feasible. Thirdly, only nine patients were performed NGS when progression, so these data cannot fully reflect the whole cohort and no statistical significance can be reached about resistance of afatinib. Despite these limitations, this study provides deep insights into clinical activity of afatinib in NSCLC with *HER2* alterations.

## Conclusion

Our results suggest that afatinib has a potential efficacy in these patients, especially in the patients with *HER2* amplification or other pathologic mutations in exons except exon 20. Further studies, especially prospective studies, are warranted to investigate the clinical activity of afatinib and the mechanism of resistance to HER2-targeted therapy.

## Data Availability Statement

The original contributions presented in the study are included in the article. Further inquiries can be directed to the corresponding authors.

## Ethics Statement

The studies involving human participants were reviewed and approved by Zhejiang Cancer Hospital. The patients/participants provided their written informed consent to participate in this study.

## Author Contributions

All authors listed have made a substantial, direct, and intellectual contribution to the work, and approved it for publication. ZS and QL contributed to the study design. All authors were responsible for interpretation of the results. ZS and SC contributed to statistical analysis. ZS and SC prepared the manuscript. All authors contributed to the article and approved the submitted version.

## Conflict of Interest

Authors SC and GW were employed by company 3D Medicines Inc.

The remaining authors declare that the research was conducted in the absence of any commercial or financial relationships that could be construed as a potential conflict of interest.

## Supplementary Material

Supplementary Figure 1 Next-generation sequencing result showed a *HER2* Y772_A775dup mutation. The BAM file was viewed using integrative genomics viewer.

Supplementary Figure 2 Kaplan-Meier estimates of progression-free survival and overall survival according to according to *HER2* mutations. **(A)** progression-free survival; **(B)** overall survival.

Supplementary Figure 3 Kaplan-Meier estimates of progression-free survival and overall survival according to lines of treatment. **(A)** progression-free survival; **(B)** overall survival.
