# Supplementary figures and images for: Efficacy and Resistance of Afatinib in Chinese Non-Small Cell Lung Cancer Patients With HER2 Alterations: A Multicenter Retrospective Study
Source: Front Oncol. 2021 May 7;11:657283. doi: 10.3389/fonc.2021.657283 (PMC8138059; doi:10.3389/fonc.2021.657283)

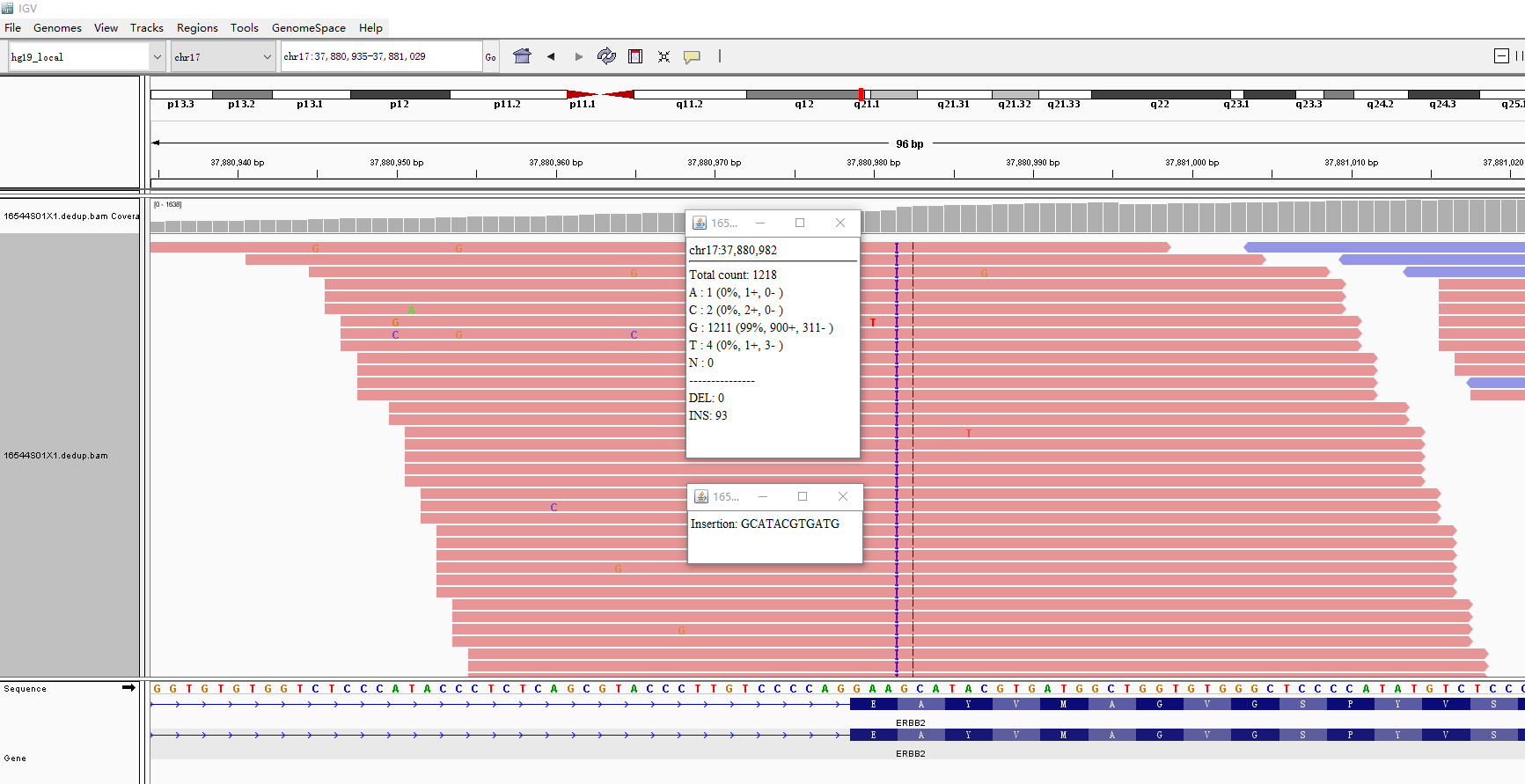

Supplement: Supplementary Figure 1 — Next-generation sequencing result showed a HER2 Y772_A775dup mutation. The BAM file was viewed using integrative genomics viewer. [file Image_1.png]

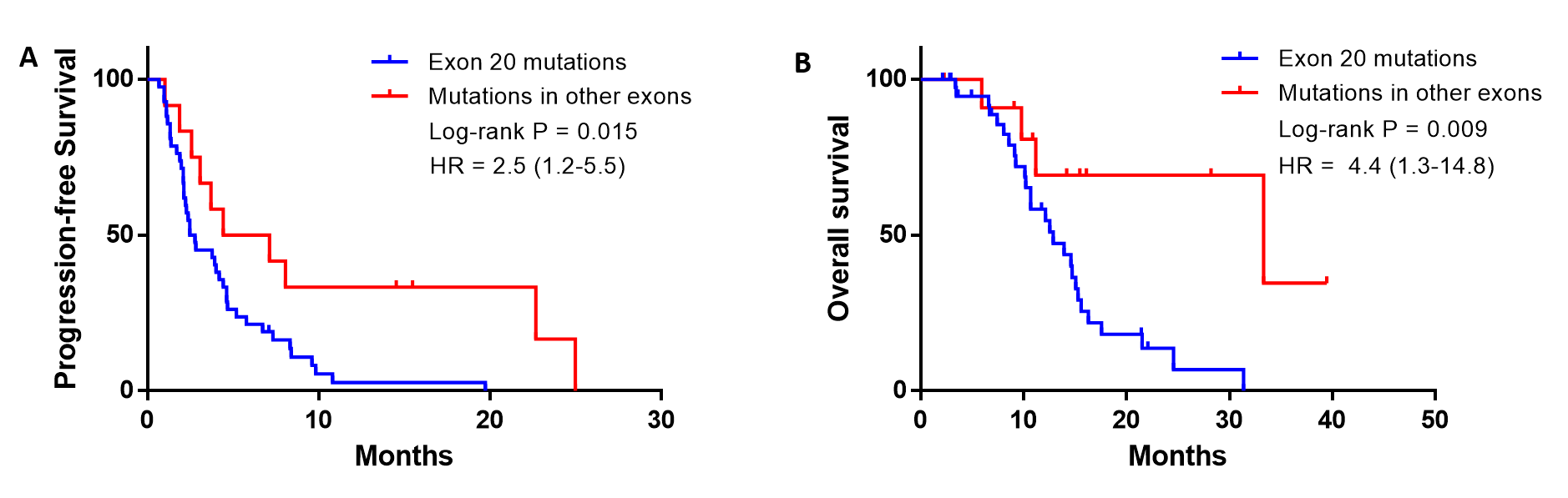

Supplement: Supplementary Figure 2 — Kaplan-Meier estimates of progression-free survival and overall survival according to according to HER2 mutations. (A) progression-free survival; (B) overall survival. [file Image_2.tif]

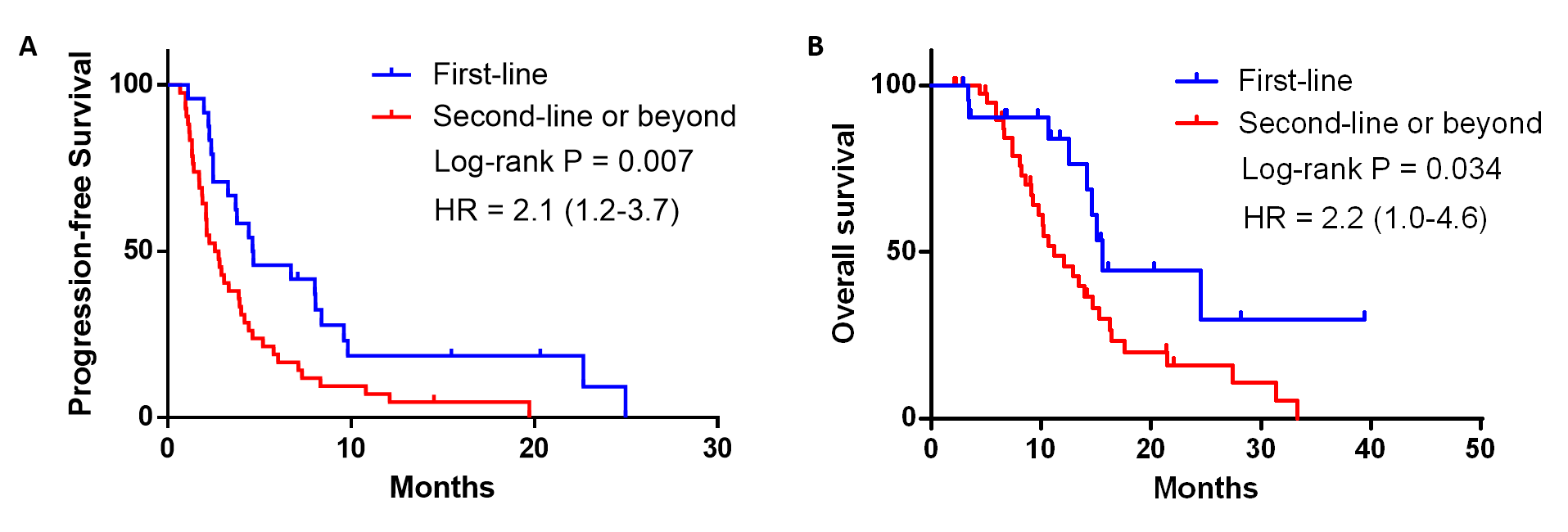

Supplement: Supplementary Figure 3 — Kaplan-Meier estimates of progression-free survival and overall survival according to lines of treatment. (A) progression-free survival; (B) overall survival. [file Image_3.tif]
