# Supplementary material for: Efficacy and Resistance of Afatinib in Chinese Non-Small Cell Lung Cancer Patients With HER2 Alterations: A Multicenter Retrospective Study
Source: Front Oncol. 2021 May 7;11:657283. doi: 10.3389/fonc.2021.657283 (PMC8138059; doi:10.3389/fonc.2021.657283)
Supplement: Supplementary file 4 [file Table_1.docx]

**Table S1. Univariate and multivariate analyses of progression-free survival**

| Parameter | | Univariate analysis | | | Multivariate analysis | | |
| --- | --- | --- | --- | --- | --- | --- | --- |
|  |  | HR | 95% CI | P | HR | 95% CI | P |
| Age |  |  |  |  |  |  |  |
|  | ≥60 vs <60 | 0.64 | 0.94-2.63 | 0.086 | 0.78 | 0.45-1.32 | 0.35 |
| Sex |  |  |  |  |  |  |  |
|  | Male vs. female | 0.86 | 0.50-1.47 | 0.582 |  |  |  |
| Smoking status, n (%) | |  |  |  |  |  |  |
|  | Yes vs. No | 0.86 | 0.50-1.52 | 0.606 |  |  |  |
| ECOG performance status, n (%) | |  |  |  |  |  |  |
|  | 2 vs. 0-1 | 1.88 | 0.88-4.00 | 0.103 | 1.58 | 0.66-3.77 | 0.3 |
| Histology, n (%) | |  |  |  |  |  |  |
|  | Squamous carcinoma vs. Adenocarcinoma | 0.31 | 0.08-1.28 | 0.105 |  |  |  |
| Brain metastasis | |  |  |  |  |  |  |
|  | Yes vs. No | 1.92 | 0.97-3.85 | 0.062 | 2.25 | 1.10-4.57 | 0.026 |
| Afatinib treatment line | |  |  |  |  |  |  |
|  | ≥2 vs. 1 | 2.11 | 1.22-3.65 | 0.007 | 2.03 | 1.13-3.65 | 0.017 |
| HER2 aberrations | |  |  |  |  |  |  |
|  | Amplification vs. mutation | 0.96 | 0.50-1.84 | 0.893 | 0.72 | 0.35-1.51 | 0.388 |
