# Supplementary material for: Efficacy and Resistance of Afatinib in Chinese Non-Small Cell Lung Cancer Patients With HER2 Alterations: A Multicenter Retrospective Study
Source: Front Oncol. 2021 May 7;11:657283. doi: 10.3389/fonc.2021.657283 (PMC8138059; doi:10.3389/fonc.2021.657283)
Supplement: Supplementary file 5 [file Table_2.docx]

**Table S2. Univariate and multivariate analyses of overall survival**

| Parameter | | Univariate analysis | | | Multivariate analysis | | |
| --- | --- | --- | --- | --- | --- | --- | --- |
|  |  | HR | 95% CI | P | HR | 95% CI | P |
| Age |  |  |  |  |  |  |  |
|  | ≥60 vs <60 | 0.75 | 0.39-1.44 | 0.388 | 1.29 | 0.60-2.78 | 0.51 |
| Sex |  |  |  |  |  |  |  |
|  | Male vs. female | 1.22 | 0.65-2.32 | 0.537 |  |  |  |
| Smoking status, n (%) | |  |  |  |  |  |  |
|  | Yes vs. No | 1.44 | 0.74-2.78 | 0.281 |  |  |  |
| ECOG performance status, n (%) | | |  |  |  |  |  |
|  | 2 vs. 0-1 | 3.26 | 1.41-7.58 | 0.006 | 3.22 | 1.02-10.11 | 0.046 |
| Histology, n (%) | |  |  |  |  |  |  |
|  | Squamous carcinoma vs. Adenocarcinoma | 0.71 | 0.17-2.98 | 0.642 |  |  |  |
| Brain metastasis | |  |  |  |  |  |  |
|  | Yes vs. No | 1.3 | 0.56-3.01 | 0.534 | 1.35 | 0.57-3.21 | 0.497 |
| Afatinib treatment line | |  |  |  |  |  |  |
|  | ≥2 vs. 1 | 2.2 | 1.04-4.63 | 0.034 | 2.09 | 0.89-4.92 | 0.09 |
| HER2 aberrations | |  |  |  |  |  |  |
|  | Amplification vs. mutation | 1.45 | 0.68-3.07 | 0.333 | 0.86 | 0.32-2.30 | 0.761 |
