# Supplementary material for: Efficacy and Resistance of Afatinib in Chinese Non-Small Cell Lung Cancer Patients With HER2 Alterations: A Multicenter Retrospective Study
Source: Front Oncol. 2021 May 7;11:657283. doi: 10.3389/fonc.2021.657283 (PMC8138059; doi:10.3389/fonc.2021.657283)
Supplement: Supplementary file 6 [file Table_3.docx]

**Table S3. *HER2* status and recurrent mutated genes in Afatinib-resistant patients**

| ID | *HER2* status before afatinib treatment | *HER2* status after progression on afatinib treatment | Mutations after progression on afatinib treatment | PFS, month | Best response |
| --- | --- | --- | --- | --- | --- |
| 1 | Amplification | Amplification | *EGFR* (E746_A750del)  *TP53* (G245C) | 2.9 | SD |
| 2 | Amplification | Amplification | *EGFR* (L858R)  *PIK3CA* (E545K) | 1.4 | PD |
| 3 | Amplification | p.G776delinsLC | *TP53* (K132Dfs*16) | 3.3 | SD |
| 4 | G776delinsLC | Y772_A775dup | *RB1* (R556*) *TP53* (R282W) | 10.8 | SD |
| 5 | G778_P780dup | p.G778_P780dup | *TP53* (c.994-2A>G) | 2.8 | PD |
| 6 | Y772_A775dup | Y772_A775dup, Amplification | *EGFR* (amplification)  *NRG1* (amplification) | 1.3 | PD |
| 7 | R896G | R896G | N/A | 14.5 | PR |
| 8 | G327E | N/A | *NRAS* (G60V) | 1.9 | PD |
| 9 | G776delinsLC | N/A | N/A | 4.4 | SD |

*EGFR*, epidermal growth factor receptor; *HER2*, Human epidermal growth factor receptor 2; mPFS, median progression-free survival; *NRAS*,NRAS proto-oncogene, GTPase; *NRG1*, neuregulin 1; PD, progression disease; *PIK3CA*, phosphatidylinositol-4,5-bisphosphate 3-kinase catalytic subunit alpha;PR, partial response;*RB1*,RB transcriptional corepressor 1; SD, stable disease; *TP53*,tumor protein p53.
